# The Attitude of Work-Oriented and Family-Oriented Chinese Women Toward the Evaluations Based on the Traditional Positive Stereotype That Women Are Virtuous

**DOI:** 10.3389/fpsyg.2021.653234

**Published:** 2021-07-28

**Authors:** Jingjing Song, Junnan Li, Yanfen Liu, Yifan Ruan

**Affiliations:** ^1^Institution of Psychology, China University of Geosciences, Wuhan, China; ^2^School of Marxism, China University of Geosciences, Wuhan, China

**Keywords:** gender role, stereotype, positive stereotypical evaluation, family-oriented, virtuous evaluation

## Abstract

People typically reject being negatively stereotyped but overlook the ways in which they are positively stereotyped. The current study focused on the attitude of Chinese women toward being evaluated based on the traditional positive stereotype that women are virtuous; family/work centrality as a boundary condition of these attitudes; and three perceptions that may mediate the link between this type of evaluation and attitudes of women. In experiment 1, female college students were identified as work-oriented or family-oriented based on their responses to a questionnaire regarding their focus on these two domains. They then read a vignette in which a man evaluated a female target under random assignment to one of three conditions, namely: group positive stereotype evaluation, individual positive stereotype evaluation, or unstereotypical positive evaluation. The participants rated how much they liked the female target, as an indicator of their attitude toward evaluations based on the stereotype that women are virtuous. In experiment 2, female college students were classified as work- oriented or family-oriented, and then read a vignette in which a man (the target) evaluated them. They were randomly assigned to the group positive stereotype evaluation, individual positive stereotype evaluation, or unstereotypical positive evaluation. Participants rated how much they liked the male target, as an indicator of their attitude toward evaluations based on the positive stereotype that women are virtuous. Across both studies, ANOVA showed that work-oriented women liked evaluations based on both group and individual stereotypes less than the family-oriented women. Regression-based analyses showed evidence of a mediation process in which work-oriented women viewed the virtuous positive stereotype as implying a prescriptive social demand that women should engage in family roles, resulting in a negative reaction to this type of evaluation.

## Introduction

The rigid but positive perception of social group members is referred to as a positive stereotype, whereby group members are perceived to have an advantage in a particular area because of their group identity (Czopp et al., 2015). Previous studies have focused on the impact of negative stereotypes on the performance of stigmatized groups and interventions to minimize this impact (Song et al., 2017), whereas lesser attention has been paid to the impact of positive stereotypes. As people view positive stereotypes as positive evaluations, the targets of these stereotypes often overlook that they are being stereotyped and depersonalized (Alt et al., 2019). This study focused on the attitudes of Chinese women toward evaluation based on the traditionally positive stereotype of women as virtuous; family/work centrality as a boundary condition of their attitudes toward this type of evaluation; and internal mechanisms of the association between this type of evaluation and the attitude. Understanding the attitude of family/work-oriented women will contribute to a deeper understanding of attitudes toward the gender roles in society of today in China and provide insight into the conditions and mechanisms of stereotype internalization.

### Evaluation of Women in China Based on the Stereotype of Women as Virtuous

Social role theory holds that social role stipulates typical characteristics commonly held by women versus men, and these social roles demand that women and men in daily life show different characteristics and behaviors based on gender (Eagly, 1987; Eagly et al., 2000; Johannesenâ-Schmidt and Eagly, 2002). People are socialized to enact these roles, internalize traditional gender stereotypes and gender schemes, have typical in-group personality characteristics, and act in accordance with their social roles. While people apply gender stereotypes to judge others women and men are stereotypically perceived as having typical feminine and masculine characteristics, respectively (Solbes-Canales et al., 2020).

A review of the literature on positive stereotypes leads us to conclude that there are three common traits ascribed to women, namely: (1) warmth-related traits, based on the stereotype content model (Fiske et al., 2002; Fiske and Taylor, 2007), (2) typical feminine traits, such as being gentle (Eagly, 1987; Eagly et al., 2000; Johannesenâ-Schmidt and Eagly, 2002), and (3) traits related to the family role, which in China includes being virtuous (Zuo, 2016). Research has also shown that women react negatively to evaluations of their professional skills based on positive stereotypes of women as cooperative and nourishing (Kervyn et al., 2012; Siy and Cheryan, 2013). To our knowledge, no research has explored attitude of women toward virtuous evaluations. In the current study, we focused on the traditional Chinese positive stereotype that women are virtuous, a characteristic with strong associations with being feminine and family-oriented.

The word “virtuous” (贤惠, “xianhui” in Chinese) is defined by the New Chinese dictionary as an adjective used to describe people who are kind, gentle, and reasonable. According to the previous literature and the common understanding that people have in the China of today. Being virtuous also refers to being family-oriented, and a virtuous woman is gentle, caring, understanding, and considerate, cares for her family, and is skilled at raising children, doing housework, and cooking. She takes care of her spouse and shows filial respect to the aging parents of her spouse (Zuo, 2016). Previous research has shown that the term virtuous is a typical stereotypical word used to describe women both in the United States and in China (Eagly, 1987; Fiske, 2010; Zuo, 2016; Eagly et al., 2000). In both cultures, women are more likely than men to be the caregivers of children and aging parents, and are more likely to exhibit traditionally feminine behaviors such as being nurturing, relation-oriented, and concerned about family (Vogel et al., 2003).

In China, being virtuous has traditionally been considered a positive trait for women. However, with the rise of gender consciousness, social roles of women have changed gradually as women pursue work, professional development, and self-fulfillment (Chang et al., 2011; Vandello et al., 2013). The meaning of the word “virtuous” as tied to the domestic roles of women is changing, and not all women in China of today perceive it as a positive term. Some women in China also have negative attitudes about being evaluated based on the stereotype that they are virtuous. Thus, our study focused on the attitudes of Chinese women toward being evaluated based on this traditional positive stereotype. We address two general questions: Do work-oriented and family-oriented Chinese women have the same reactions to evaluations based on the traditional positive stereotype of women as virtuous? What factors explain the connection between this type of evaluation and the reactions of women to them?

### Attitude of People Toward Being Evaluated Based on Positive Stereotypes

Evaluations based on positive stereotypes are not as noticeable as those based on negative stereotypes, as people tend to think of them as friendly compliments rather than evidence of prejudice or discrimination (Barreto and Ellemers, 2005; Mae and Carlston, 2005; Czopp et al., 2015). For example, positive stereotypes of women are often perceived as flattering rather than hostile (Glick and Fiske, 1996; Jost and Kay, 2005). Expressions of positive stereotypes can be perpetuated because people like those who compliment them (Gordon, 1996) and generally prefer to be seen in positive light (Schlenker, 1980). Thus, people accept and apply many positive stereotypes, and these evaluations can have a positive impact on interpersonal relationships (Jost and Kay, 2005).

At the same time, many studies have pointed out that people sometimes dislike being evaluated based on positive stereotypes, just as they dislike being evaluated based on negative stereotypes. In particular, people dislike being evaluated based on positive group stereotypes (“women are nurturing”) compared with being evaluated based on positive stereotypes at the individual level (“you are nurturing”) (Garcia et al., 2006; Siy and Cheryan, 2013, 2016), being evaluated based on non-stereotypical positive evaluations (“you are good”) (Kervyn et al., 2012), and not being evaluated at all (Czopp, 2008; Siy and Cheryan, 2013). Research on this topic has mainly focused on positive stereotypes about race (Africans have high athletic ability, Asians are good at math, hardworking, and ambitious) and gender (female are warmth, cooperative/nurturing, good at dance). In the current study, we focused on gender using information collected from a sample of women in China.

Contradictory results related to the attitude toward positive stereotyped evaluation are likely due to the following two reasons. First, the reference standards are different. The idea that people liked positive stereotype evaluation mainly compared the attitude toward the negative stereotyped evaluation with positive stereotyped evaluation (Czopp et al., 2015). But the studies that found people reject group positive stereotyped evaluation compared positive evaluation with unstereotypical positive evaluation (Kervyn et al., 2012). Therefore, different reference criteria may be one of the reasons for the contradictory results.

Second, characteristics, identities, and motivations of people will affect their attitude toward positive stereotyped evaluation. Scholars also point out that not everyone rejects positive stereotyped evaluation, and there exist important moderators (Siy and Cheryan, 2013). For example, it has been demonstrated that, for those who engage in individualistic cultures, being the target of a positive stereotype, which indicated been perceived as a group member, may be in direct conflict with the desire to define the core self of an individual as a separate and distinct entity, and thus they dislike much more the positive stereotype evaluation (Siy and Cheryan, 2013). However, for those who engage in collectivistic cultures, being the target of a positive stereotype, which indicated been perceived as connected to other group members, is more compatible with how they see themselves, and thus they may dislike less the positive stereotype evaluation (Siy and Cheryan, 2013).

### Family/Work Centrality as a Moderator of Attitudes of Women Toward Evaluations Based on the Positive Stereotype of Being Virtuous

In order to further understand ambivalent attitudes of people toward positive stereotypical evaluations, what kinds of people reject positive stereotypical evaluations was explored. In the current study, we focused on an individual difference variable as a moderator of the association between evaluations based on positive stereotypes and the attitude of women toward those evaluations. The individual difference of interest is whether women are work-oriented or family-oriented.

Social role theory provides the conceptual framework for thinking about these stereotypes about gender. This theory holds that society has different role requirements for people of different genders (Henry et al., 2013). A common stereotype is that women should be family-oriented, with the welfare of the family being central to their lives; men should be work-oriented, meaning that they should value development, continuous learning, and gaining experience (Chang et al., 2010; Beigi et al., 2017). Carr et al. (2008) put forward the concept of work–family centrality, which refers to a value judgment about the relative importance of the work role versus family role, with the work-oriented and family-oriented as the two ends of this continuous variable (Xie et al., 2017).

We assumed that attitude of family-oriented women toward virtuous evaluation was more positive than work-oriented women. Family-oriented women think of family as a key aspect of their identity, value family relationships, internalize the women identity, tend to follow gender role norms for raising children, do housework and cleaning. thus, we suspect that they are fond of being praised for being virtuous. Work-oriented women who value work outside the home and pursue personal career development would think the attributes and behaviors related to a family role less important. Work-oriented women, compared to family-oriented women, may spend less time and energy performing household duties and caring for the family. Thus, being evaluated based on the stereotype that women are virtuous may be inconsistent with their values. Work-oriented women may resist and feel angry about an evaluation based on this stereotype, as it might carry with its implicit expectations that they should put family first, which is in conflict with the values they place on work. In sum, work versus family-orientation should moderate the association between evaluations based on the “virtuous” positive stereotype and attitudes of women toward such evaluations.

### Mechanisms Underlying Attitude of People Toward Being Evaluated Based on Positive Stereotypes

Differ with the mechanism of people reject positive stereotypes, people angry of been negative stereotypes evaluation is mainly because of self-identity, self-affirmation, and self-protection needs, and thus don’t like the negative evaluation. According to the intergroup emotion theory, negative comments, either personal insults or group slurs, should elicit negative emotions such as anger and behavioral action tendencies such as the desire to confront the speaker (Garcia et al., 2006). One question concerns the reason for (or the mechanism of) the dislike of group-stereotypic positive comments by the people. Evaluations based on stereotypes afford a series of inferences, namely: evaluators hold stereotypes about the group of a person; they use a within-category judgment standard to judge members of the group (Biernat et al., 1997); they expect group members to act in accordance with stereotypes; they believe that counter-stereotyped behavior should be punished. These inferences would result in a negative reaction to evaluations based on stereotypes, even positive stereotypes.

One mediator of the association between evaluations based on positive stereotypes and the attitude of people toward these evaluations may be the perception of depersonalizing. That is, people may reject even positive stereotypical evaluations because such evaluations make them feel that the evaluator focuses only on their group identity and does not take into account their personal characteristics and uniqueness (Siy and Cheryan, 2013). A study by Siy and Cheryan confirmed that positive stereotypical comments about women by unfamiliar evaluators can make women feel depersonalized, which in turn can lead to female participants disliking the evaluator (Siy and Cheryan, 2013). Thus, depersonalization of the target may mediate the association between evaluations based on positive stereotypes and attitudes toward the evaluations.

Another mediator of this association may be the perception of people that the stereotypically positive trait implies the target also has stereotypically negative traits. According to models of spreading activation, the information networks about a social group are intertwined and linked in their memory system (Neely, 1976). As long as one stereotype is activated, another stereotype about the same group will be activated and affect the social judgment (Kay et al., 2013). Kervyn et al. (2012) called this the innuendo effect. When evaluators are given information about the stereotypically positive traits of a person on a first dimension, they tend to perceive that the person being evaluated as stereotypically negative on another dimension. This complementary effect has been demonstrated in several studies (Abele and Wojciszke, 2007; Fiske and Taylor, 2007; Kervyn et al., 2012). For example, when women exhibit stereotypically positive traits and behaviors, such as warmth and friendliness, in the work environment, they also tend to be assessed as showing stereotypically negative traits such as being less competent and less capable of leading than men (Williams and Tiedens, 2015; Brescoll, 2016). In summary, the perception of the target as having both stereotypically positive and stereotypically negative traits (in the current study, being virtuous and being incompetent, respectively) could mediate the association between the evaluation and attitude toward the evaluation.

A third mediator may be the perception that positive stereotypes are prescriptive, especially when compared with stereotype-based praise on a personal level (Czopp et al., 2015). Evaluation based on the “virtuous” stereotype for Chinese women may convey an implicit expectation that women should be engaged in activities associated with their family roles as daughter, spouse, and mother. Therefore, we think that the views of a woman about whether being virtuous conveys an expectation to engage more with the family and home may mediate the association between an evaluation based on the virtuous stereotype and the attitude of women about the evaluation.

In the current study, we examined three perceptions, namely: the depersonalization perception, the low competence evaluation perception, and the family role perception as possible mediators to explain why some women dislike the evaluation of being virtuous. These mediators could likely be applied to understanding the negative reactions of people to negative stereotypes as well, but we will explain our reasoning with regard to the focus of the current study, which is the traditionally positive stereotype that Chinese women are virtuous.

### The Present Study

This study was conducted to test whether Chinese work-oriented and family-oriented women have different attitudes toward evaluations based on the traditionally positive stereotype of women as virtuous. It also tested the mediation role of depersonalization perception, low competence evaluation perception and family role perception in the relation between the positive stereotype evaluation, and the attitude of women toward this evaluation. Two experiments were conducted on samples of women in China from the actor and observer perspective. Using a between-group design, participants in each experiment were randomly assigned to read one of three vignettes and then rate one of the characters. In Experiment 1 (observer perspective) participants read a vignette in which a male character evaluated the female target as virtuous based on a group stereotype, evaluated her as virtuous as an individual trait, and provided a positive evaluation unrelated to the virtuous stereotype. The participants then rated how much they liked the female target and were willing to be like her. In Experiment 2 (actor perspective) participants were randomly assigned to read a vignette in which the participants themselves were evaluated by a man, using evaluations similar to those in Experiment 1. Afterward, they rated the likeability of the male target who evaluated them in the vignette. The three dependent measures (likeability of the female target, willingness to be like the female target, and likeability of the male evaluator) were used to indicate the attitudes of a participant about evaluations based on the Chinese traditionally positive stereotype that women are virtuous. Three perceptions such as: the perception that this type of evaluation creates depersonalization, implies negative stereotypes, and prescribes engagement in family roles were tested as mediators of the association between this type of evaluation and the attitudes of women toward these evaluations:

## Experiment 1

The purpose of Experiment 1 was to explore the attitude of female participants toward a female target who has been evaluated based on the positive stereotype that women are virtuous; the participants took the perspective of an observer. We analyzed whether work-oriented women disliked the virtuous female target more than family-oriented women did, and in the subsample of work-oriented women, we tested the mediation roles of the depersonalization of the target, the perception that the target also showed the negative stereotype of being incompetent, and the perception that virtuous women are engaged in family role responsibilities. In a between-subjects design, the female participants were randomly assigned to read one of three vignettes, using the observer perspective. The vignettes described a female target who was evaluated by a male evaluator (1) based on a positive stereotype at the group level (“like most women, she is virtuous”), (2) based on a positive stereotype at the individual level (“she is virtuous”), and (3) based on a general positive trait (she is good). The degree of “likability of the target” and “willingness to be with a women like the target” were used to reflect the attitude of positive stereotyped evaluations.

### Method

#### Participants

We recruited a total of 168 women who were students at a university in central China. After excluding four participants who gave highly consistent ratings on all items, the retained sample comprised 164 participants. These age of the participants ranged from 18 to 33 years (*M* = 21.36, *SD* = 2.50). There were 74 from rural areas (45.1%) and 90 from urban areas (54.9%). They were all unmarried with no children.

An *a priori* power analysis was conducted with G^∗^Power to determine the required number of participants for testing ANOVA main effects and interactions, with alpha at 0.05, power at 80%, and effect size f at 0.3 (Faul et al., 2009). The required sample size was determined to be *N* = 149.

#### Procedure

Permission to conduct the experiment was granted by the Research Ethics Committee at the institution with which the first author was affiliated. The purpose and requirements of the study were explained, and then participants gave written informed consent to participate. They then provided demographic information and completed the family/work centrality questionnaire. Next, the participants were randomly assigned to one of the three conditions: group positive stereotype evaluation, individual positive stereotype evaluation, or control. In each condition, the participants read a vignette in which people should evaluate one women target. The woman who was chosen was described by a male evaluator in three different ways, depending on the condition. The participants then rated how much they liked the target and how willing they were to be like the target. Finally, they completed questionnaires concerning their perceptions of depersonalization, the implied negative stereotype of women as incompetent, and the expectation of engagement in family roles. When the study was complete, the participants were given a small reward to thank them for their help.

#### Measurement

##### Participants’ family/work centrality

We measured the family/work centrality of the participants using a questionnaire designed for the current study. The measure contained six items: (1) I will put family first and work second in the future. (2) I will spend more time and energy taking care of my family than pursuing my career development in future. (3) I will spend more time taking care of my children and husband than focusing on my hobbies and interests in the future. (4) I will put work first and family second in the future. (5) I will spend more time and energy pursuing my career development and achievement than taking care of my family in the future. (6) I will spend more time focusing on my hobbies and interests than taking care of my children and husband in the future. A seven-point rating scale was used, with 1 = *completely inconsistent with my thinking*, and 7 = *completely consistent with my thinking*. The last three questions were scored in reverse. The sum of the six items scores was the final score, with high scores indicating a tendency toward having a family orientation and low scores indicating a tendency toward having a work orientation. In our study, those participants with a score above the median were considered family-oriented (*N* = 96), while those with a score below the median were considered work-oriented (*N* = 68). The internal consistency (α) of this questionnaire was 0.75 in the current study.

##### Manipulation of positive stereotyped evaluation

Participants were randomly assigned to read one of three vignettes. The vignettes were based on earlier research (Kervyn et al., 2012). Specifically, participants read a vignette in which a male peer described a female target. The description of the female target by a male peer in the group stereotyping condition was “just like most women, she is very virtuous.” The description in the individual positive stereotyping evaluation condition was “she is very virtuous.” The description in the control condition was simply “she is good.” We measured how much the participants liked the female target (likeability) and their willingness to be like this woman.

##### Likeability of the target

To assess how much the participants saw the female target as likable, we asked participants to rate two items to indicate how much they liked the target and how willing they were to be friends with the target using a seven-point thermometer scale. The items were “Do you like the target?” (1 = *dislike the target very much*, 7 = *like the target very much*) and “Please describe your degree of willingness to be friends with the target” (1 = *do not want to make friends with the target at all*, 7 = *very much want to make friends with the target*). This method has been used in previous research (Shelton et al., 2005; Czopp, 2008), and it was translated into Chinese from English. The sum of the two items was the final score. Higher scores indicated higher likeability of the target. The internal consistency (α) of this questionnaire in the current study was 0.75.

##### Willingness to be a woman like the target

We assessed how willing the participants were to be like the target, using the question, “How much are you willing to be a woman like her?” A seven-point scale was used (1 = *not at all*, 7 = *very much*). The higher the score, the higher the willingness to be like the target. This variable was used to indirectly reflect the attitude of the participants toward evaluations based on the positive stereotype of women as virtuous. When participants in the positive group stereotype condition reported lower willingness to be a woman like the target than participants in the control condition, which referred that female dislike to be a woman who have been evaluated based on the positive stereotype that women are virtuous, we inferred that they were resistant to being evaluated based on the positive stereotype.

##### Depersonalization of the target

We assessed depersonalization of the target with four questions that were translated into Chinese from English (Siy and Cheryan, 2013). The items were “I see this woman as more than just a member of her gender group,” “I would judge this woman based solely on her gender,” “To what extent does this woman is identical to other members of her gender group,” “To what extent does I see this woman only for her gender group.” Participants made their ratings on a seven-point scale from 1 (*not at all*) to 7 (*very much*). A higher score indicated higher depersonalization of the target by the participant. The internal consistency (α) of this questionnaire in the current study was 0.51.

##### Evaluation of the competence of the target

We measured the competence evaluation of the target by the participant by asking them to rate three trait words (competent, efficient, and enterprising) in terms of how well they described the target. Each trait was rated using a Likert scale from 1 (*not at all descriptive*) to 5 (*very descriptive*). The sum of the three items was the final score. The higher the score, the higher the perceived competence of the target. The internal consistency (α) of this questionnaire in the current study was 0.73. This method has been used in previous research (Kervyn et al., 2012; Song and Zuo, 2016).

##### Evaluation of the family role of the target

A self-designed six-item questionnaire was used to measure the perception of the participant on the engagement of the target in her family roles. Example items were “I think the target is family-oriented rather than work-oriented,” “I think the target is very good at housekeeping,” and “I think the target would take care of her children and her husband when she has a family.” A seven-point scale was used, with 1 = *I do not think so at all*, and 7 = *I agree with this description very much.* The higher the score, the higher the perceived engagement in family roles. The internal consistency (α) of this questionnaire in the current study was 0.75.

#### Data Analysis

We first generated the means and standard deviations of all study variables and the correlations among them. In the full sample, we then conducted two 2 (family/work centrality: family-oriented vs. work-oriented) × 3 (positive stereotype evaluation: individual vs. group vs. control) between-subjects analyses of variance (ANOVAs) to analyze the influence of these independent variables on the ratings of the likeability of the target and the willingness to be like the target. Lastly, in the subsample of work-oriented women, we used the process macro for SPSS (Model 4) for multiple mediation effect analysis (Hayes, 2012) to test the effects of the three proposed mediators of the association between type of evaluation and willingness of the women to be like the target. The three mediators were depersonalization of the target, evaluation of the competence of the target, and evaluation of the engagement in family roles of the target.

### Results

#### Correlations and Descriptive Statistics

Correlations among all study variables were calculated using Pearson product-moment correlation coefficients (see Table 1). The results indicated that the willingness to be like the target was positively associated with the evaluation of the competence of the target, and negatively associated with depersonalization of the target by the participant, and perception of the engagement in family roles of the target.

**TABLE 1:** Correlations, means, and standard deviations for all variables in Experiment 1.

	Dummy variable 1	Dummy variable 2	Centrality	Depersonalize	Competence	Family role	Likeability	Willingness to be the target
1	–							
2	−0.51***	–						
3	–0.13	0.06	–					
4	0.02	0.07	0.05	–				
5	–0.05	0.002	0.05	0.09	–			
6	0.20**	0.31***	–0.04	0.10	−0.28***	–		
7	0.07	–0.01	0.22**	0.11	0.24**	0.07	–	
8	–0.13	–0.004	0.37***	0.17*	0.29***	−0.16*	0.41***	–
*M*	0.34	0.33	21.08	14.76	15.12	27.15	9.53	3.93
*SD*	0.47	0.47	5.58	2.56	2.70	4.76	1.59	1.40

The positive stereotyped evaluation contained three experimental conditions, thus it was set as two dummy variables, in which the control group is 0, 0; the individual positive stereotyped evaluation group was 1, 0, and renamed as dummy variable 1; and the group positive stereotyped evaluation group was 0, 1, and renamed as dummy variable 2. *p* < 0.001***, *p* < 0.01**, *p* < 0.05*.

#### Influence of Family/Work Centrality and Positive Stereotype Evaluation on Evaluation of the Target

The 2 (family/work centrality: family-oriented vs. work-oriented) × 3 (positive stereotype evaluation: individual vs. group vs. control) between-subjects analysis of variance (ANOVA) with likeability as the dependent measure showed a significant main effect of family/work centrality, *F*(1,158) = 4.92, *p* = 0.03, η_*p*_^2^ = 0.03: participants who were family-oriented (*M* = 9.75, *SD* = 1.68) rated the target as more likeable than participants who were work-oriented did (*M* = 9.22, *SD* = 1.40). The main effect of positive stereotype evaluation was not significant, *F*(2,158) = 0.78, *p* = 0.46, and the interaction effect was also not significant *F*(2,158) = 0.76, *p* = 0.47.

The 2 (family/work centrality: family-oriented vs. work-oriented) × 3 (positive stereotype evaluation: individual vs. group vs. control) between-subjects analysis of variance (ANOVA) with willingness to be a women like the target as the dependent measure showed a significant main effect of family/work centrality, *F*(1,158) = 4.93, *p* = 0.03, η_*p*_^2^ = 0.03. Participants who were family-oriented (*M* = 4.16, *SD* = 1.30) reported higher willingness to be a women like the target than participants who were work-oriented (*M* = 3.62, *SD* = 1.48). The main effect of the type of positive stereotype evaluation was not significant, *F*(2,158) = 2.37, *p* = 0.09, but the interaction effect was significant, *F*(2,158) = 3.41, *p* = 0.04, η_*p*_^2^ = 0.04.

**FIGURE 1:**
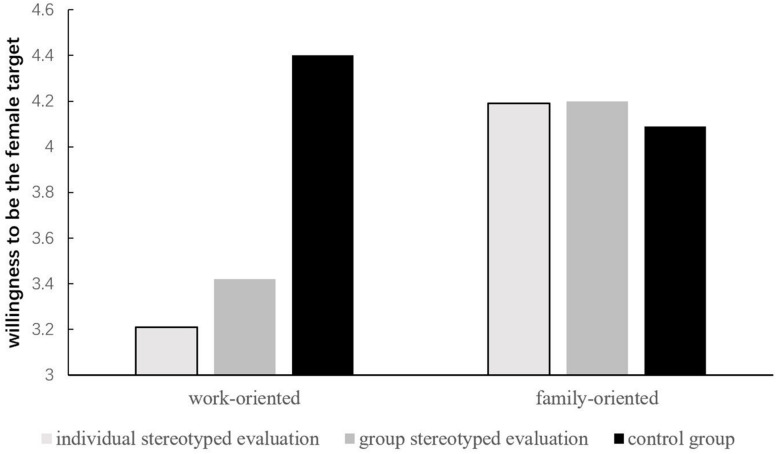
Degree of willingness to be the female target who were evaluated by their male peer based on the positive stereotype.

Simple effects analysis showed that, for the work-oriented participants, the effect of positive stereotype evaluation type was significant, *F*(2,158) = 5.59, *p* < 0.01, η_*p*_^2^ = 0.06. Work-oriented participants in the individual positive stereotype evaluation condition (*M* = 3.21, *SD* = 1.45) and the group positive stereotype evaluation condition (*M* = 3.42, *SD* = 1.50) reported lower willingness to be a woman like the target than participants in the control condition (*M* = 4.40, *SD* = 1.23). The simple effect of positive stereotype evaluation type for the family-oriented participants was not significant, *F*(2,158) = 0.07, *p* = 0.94. Thus, the results brought evidence that the work-oriented women would reject being evaluated based on the positive stereotype that women are virtuous more than family-oriented women would. Figure 1 illustrates this interaction.

#### Mediators of Rejection of Evaluations Based on the Chinese Traditional Positive Stereotype of Women as Virtuous

We further focused on the internal mechanisms of rejection of work-oriented participants toward the evaluation based on the positive stereotype of women as virtuous. Specifically, we tested the effect of positive stereotype evaluation type (the independent variable) on the willingness of the participant to be the target (the dependent variable), and tested the mediation role of the depersonalization of the target, rating of the competence of the target, and perception of the engagement in family roles. There were three conditions for the positive stereotype evaluation type, coded as two dummy variables (control condition: 0, 0; individual positive stereotype evaluation condition: 1, 0; and group positive stereotype evaluation: 0, 1). The regression analysis that tested the effect of the positive stereotyped evaluation manipulation on the “willingness to be the target” could provide information about the attitude of the participants toward evaluations based on the positive stereotype of women as virtuous. If women in the positive stereotype evaluation condition were less willing to be like the target compared with women in the control condition, it suggested that they would dislike being evaluated based on the positive stereotype of women as virtuous.

We used the process macro for SPSS (Model 4) to conduct multiple parallel mediation effect analysis (Hayes, 2012) in the subsample of work-oriented women. The analysis tested the simultaneous effects of three mediators (depersonalization of the target, evaluation of the competence of the target, and perception of the engagement in family roles of the target in the association between the type of evaluation and attitude of the participants toward the female target (willingness to be like the target, used as an indicator of attitude of the participant toward the type of evaluation). In order to test the multiple parallel mediation model, the parameters for five regression models were required to be estimated. In Model 1, the effect of independent variables on the dependent variable would be estimated. In Model 2, the effect of independent variables on mediators one would be estimated, and we focused on t mediator two and three in models 3 and 4, respectively. In model 5, the effect of the independent variable and mediators on dependent variables would be estimated. All predictors were standardized to minimize multicollinearity (Dearing and Hamilton, 2006).

Model 1 tested the relationship between the independent variable (type of positive stereotyped evaluation) and the dependent variable (willingness to be like the target). The equation was significant, *R*^2^ = 0.12, *p* = 0.01. Being in the individual positive stereotype evaluation condition and in the group positive stereotype evaluation condition both significantly predicted the willingness to be like the target, *B* = −1.19, *t* = −2.92, *p* = 0.005; *B* = −0.98, *t* = −2.17, *p* = 0.03. Participants in these conditions were less willing to be like the virtuous target compared with participants in the control condition. Thus, we inferred that work-oriented participants disliked evaluation based on the positive stereotype of being virtuous.

Model 2 tested the association between the independent variable and the first mediator (depersonalization of the female target). The equation was not significant, *R*^2^ = 0.02, *p* = 0.53. Neither being in the individual positive stereotype evaluation condition nor the group positive stereotype evaluation condition predicted the depersonalization of the target, *B* = 0.68, *t* = 0.92, *p* = 0.36; *B* = 0.85, *t* = 1.04, *p* = 0.30.

Model 3 tested the association between the independent variable and the second mediator (evaluation of the competence of the female target). The equation was not significant, *R*^2^ = 0.03, *p* = 0.31. Neither being in the individual positive stereotype evaluation condition nor in the group positive stereotype evaluation condition predicted the competence evaluation of the target, *B* = −0.91, *t* = −1.17, *p* = 0.24; *B* = −1.26, *t* = −1.47, *p* = 0.14.

Model 4 tested the association between the independent variable and the third mediator (perception of the engagement in family roles of the female target). The equation was significant, *R*^2^ = 0.33, *p* < 0.001. Being in the individual positive stereotype evaluation condition and being in the group positive stereotype evaluation condition both significantly predicted the perception of the family role of the target, *B* = 4.99, *t* = 4.44, *p* < 0.001; *B* = 5.97, *t* = 4.82, *p* < 0.001. Compared with the control group, participants in the individual positive stereotype evaluation condition and in the group positive stereotype evaluation condition were more likely to perceive the target as being engaged in family roles.

Model 5 tested the independent variable (type of positive stereotyped evaluation) and all three mediators (depersonalized perception, competence perception, and family-role perception) to predict the dependent variable (willingness to be like the female target). The equation was significant, *R*^2^ = 0.24, *p* = 0.004. The individual positive stereotype evaluation condition and the group positive stereotype evaluation condition did not predict the willingness to be like the virtuous target, *B* = −0.63, *t* = −1.42, *p* = 0.16; *B* = −0.30, *t* = −0.60, *p* = 0.55. The depersonalization of the target and the evaluation of the competence of the target, also did not predict willingness to be like the virtuous target, *B* = 0.04, *t* = 0.66, *p* = 0.51; *B* = 0.06, *t* = 0.97, *p* = 0.34. The only significant effect was for the perception of the engagement in family roles of the target. This perception negatively predicted the willingness to be like the virtuous target, *B* = −0.10, *t* = −2.32, *p* = 0.02. The work-oriented participants were less willing to be like the virtuous target, as they associated being virtuous with being engaged in family roles.

The mediating effect analysis showed that the mediation role of depersonalization in the relationship between the positive stereotyped evaluation (dummy variable 1 and dummy variable 2) and the willingness to be like the virtuous target was not significant, *B* = 0.03, *LLCI* = −0.13, *ULCL* = 0.20; *B* = 0.04, *LLCI* = −0.19, *ULCL* = 0.22. The mediation role of competence evaluation was also not significant, *B* = −0.06, *LLCI* = −0.23, *ULCL* = 0.11; *B* = −0.08, *LLCI* = −0.34, *ULCL* = 0.13. Only the mediation role of family-role perception was significant, *B* = −0.53, *LLCI* = −1.17, *ULCL* = −0.05; *B* = −0.63, *LLCI* = −1.27, *ULCL* = −0.06. Positive stereotype evaluation of the target as virtuous was associated with a higher perception that the target was family-oriented, which in turn was associated with lower willingness to be like the target for the work-oriented participants.

In summary, Experiment 1 found that work-oriented women who read a vignette about a woman evaluated by a male based on a group positive stereotype (“like every woman, she is virtuous”) or an individual positive stereotype (“she is virtuous”) is less likely to be a women like the target than participants in the control condition (“she is good”). Mediation analysis found that the perception of engagement in family roles was a significant mediator in the association between evaluations based on the positive stereotype of women being virtuous and the participants’ lower willingness to be like the target.

Experiment 1 assessed attitudes of the participants from the observer perspective, that is, how much they liked the female target who was being evaluated. In Experiment 2, we further examined attitudes of the participants from the actor perspective, that is, how much they liked the male target who was evaluating them. In Experiment 2, participants were randomly assigned to read one of the three vignettes which they were asked to imagine themselves in a hypothetical situation that they have been evaluated by a male peer, corresponding to the group-level positive stereotype, the individual-level positive stereotype, or the neutral (control) evaluation.

## Experiment 2

The purpose of Experiment 2 was to explore attitude of the participants toward the positive stereotyped evaluation of being virtuous under the actor perspective, analyzed whether work-oriented females much more dislike the male partner who evaluated them as virtuous compared with the family-oriented female, and the mediation role of being depersonalized perception, competence perception, and family role perception. Participants were asked to imagine themselves in a hypothetical situation where a male class leader assigned tasks to them, and they were randomly assigned to group positive stereotypes group, individual positive stereotypes group, and control groups through the male class leader description. Their ratings of the likeability of the evaluator were used as an indicator of their attitude toward being evaluated based on the positive stereotype that women are virtuous.

### Method

#### Participants

A total of 201 volunteers were recruited to participate. Two were excluded after they identified themselves as men, leaving a final sample of 199 participants. The age of the participants ranged from 16 to 36 years (*M* = 21.17, *SD* = 2.90). There were 80 from rural areas (40.2%) and 199 from urban areas (59.8%). They were all unmarried and with no children.

#### Procedure

After being told the purpose and requirements of the study, the participants gave their written informed consent. Participants firstly provided demographic information, and then completed the family-orientation questionnaire. Then, they were randomly assigned to read a vignette corresponding to one of three conditions: individual positive stereotype evaluation, the group positive stereotype evaluation, or control. Finally, the participants rated how much they liked the male evaluator in the vignette, and they completed questionnaires concerning their perceptions of depersonalization, the perception of incompetent evaluation, and the perception of family roles. When the study was complete, the participants were given a small reward to thank them for their help.

#### Measurement

##### Participants’ family/work centrality

We used the same measurement as in Experiment 1. Based on the median split, 101 participants were categorized as showing a work-oriented tendency, and 98 participants were categorized as showing a family-oriented tendency.

##### Manipulation of positive stereotype

We adjusted the vignettes used in previous studies according to the life circumstances of female college students (Siy and Cheryan, 2013). We asked participants to imagine a situation in which two classes have a party together. In this party, the male class leader assigned the participant to help with cooking or to help with the physical work of preparing the site. The different expressions of the male class leader were used to manipulate positive stereotyped evaluation. In the individual positive stereotype evaluation condition, the male class leader said, “You are very virtuous. Could you cook a meal?” In the group positive stereotype evaluation condition, the male class leader said, “I know all women are so virtuous. Could you cook a meal?” In the control condition, he just said, “Could you cook a meal?” Participants were randomly assigned to one of the three conditions.

##### The likeability of the male class leader

Please refer to Experiment 1. The internal consistency of this questionnaire (α) in the current experiment was 0.91. This variable was used to indirectly reflect the attitude of the participants toward being evaluated based on the stereotype that women are virtuous. When the likeability of the male class leader was lower in the positive stereotype evaluation conditions than in the control condition, it indicated that the participants were more resistant to being evaluated based on the positive stereotype that women are virtuous.

##### Being depersonalization perception

We used four items that were translated to Chinese from English to assess the perception of being depersonalized by male class leaders (Siy and Cheryan, 2013). The items were, “The male class leader sees me only for your gender group,” “The male class leader sees me as more than just a member of your gender group,” “The male class leader judged me based solely on my gender,” “The male class leader thinks I am identical to other members of my gender group.” A seven-point Likert scale was used, with 1 = *not at all*, and 7 = *very much*. The higher the score, the higher the perception of being depersonalized. The internal consistency (α) of this questionnaire was 0.56.

##### Perception of the competence evaluation of the male class leader toward the participant

We used three trait words (competent, efficient, and enterprising) to measure the perception of the competence evaluation of the male class leader toward the participant (Kervyn et al., 2012; Song and Zuo, 2016). For example, *I think, in the mind of this male class leader, I am competent*. A five-point Likert scale was used, with 1 referring to *not at all*, and 5 referring to *very much*. The sum of the three items was the final score representing overall competence. The higher the score was, the higher the perceived evaluation of competence was. The internal consistency (α) of this questionnaire in the current study was 0.83.

##### Perception of family role engagement evaluation that male class leader toward the participant

A six-item self-designed questionnaire was used to measure the perception of family role engagement evaluation that male class leader toward the participant (e.g., *I think, in the mind of male class leader, I am family-oriented*). A seven-point Likert scale was used, with 1 = *not at all*, and 7 = *very much.* The sum score of these six items was the final score; the higher the score was, the higher the family-role perception. The internal consistency (α) of this questionnaire in the current study was 0.83.

### Results

#### Correlations and Descriptive Statistics

The correlations among all study variables were calculated using the Pearson’s product-moment correlation coefficient (see Table 2). The results indicated that the likeability of the target was positively associated with the perception of the competence evaluation, and negatively associated with the perception of being depersonalized and the perceived family role.

**TABLE 2:** Correlations, means, and standard deviations for all study variables in Experimental 2.

	Dummy variable 1	Dummy variable 2	Centrality	Depersonalized	Competence	Family-role	Likeability
1	–						
2	−0.50***	–					
3	0.03	–0.05	–				
4	–0.07	0.14	–0.09	–			
5	–0.01	–0.01	0.15*	−0.25***	–		
6	0.05	0.17*	–0.02	0.36***	−0.22**	–	
7	–0.002	0.02	0.35***	−0.44***	0.43***	−0.32***	
*M*	0.29	0.38	19.26	26.94	12.32	30.42	4.94
*SD*	0.45	0.49	5.72	4.44	3.42	5.89	2.40

*p* < 0.001***, *p* < 0.01**, *p* < 0.05*.

#### The Influence of Family/Work Centrality and Type of Positive Stereotype Evaluation on Attitude of the Participants Toward Being Evaluated Based on Positive Stereotype Evaluation

We conducted a 2 (Family/work centrality: family-oriented vs. work-oriented) × 3 (positive stereotyped evaluation: individual vs. group vs. control) between-subjects analysis of variance (ANOVA) with likeability of the male class leader as the dependent variable. The results showed that the main effect of family/work centrality was significant, *F*(1,193) = 13.43, *p* < 0.001, η_*p*_^2^ = 0.06. Participants who were family-oriented (*M* = 5.51, *SD* = 2.43) reported liking the target more than participants who were work-oriented (*M* = 4.38, *SD* = 2.25). The main effect of evaluation type was not significant, *F*(2,193) = 0.18, *p* = 0.84, but there was a significant interaction, *F*(2,193) = 4.14, *p* = 0.02, η_*p*_^2^ = 0.04.

**FIGURE 2:**
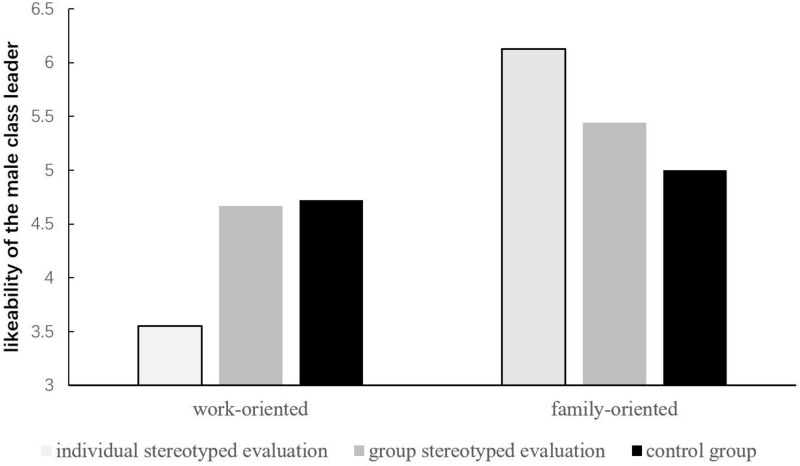
Likeability of the male leader who evaluated the women base on the positive stereotype by work-oriented and family-oriented women.

The simple effects analysis showed that the effect of family/work centrality was significant in the individual positive stereotype evaluation condition, *F*(1,193) = 17.83, *p* < 0.001, η_*p*_^2^ = 0.08. Participants who were family-oriented (*M* = 6.13, *SD* = 3.05) reported higher likeability of the male class leader than participants who were work-oriented (*M* = 3.55, *SD* = 2.04). The simple effect of family/work centrality was not significant in the group positive stereotyped evaluation group, *F*(1,193) = 1.99, *p* = 0.16, *F*(1,193) = 0.24, *p* = 0.63. Our results suggest that only the worked-oriented women disliked been evaluated as virtuous. See Figure 2.

#### Mechanism of Rejection Attitude of Being Evaluated Based on Positive Stereotype for the Work-Oriented Participants

We tested three mediators of the association between positive stereotype evaluations and the attitude of work-oriented participants toward this type of evaluation. The experimental manipulation (coded as dummy variables) of the positive stereotyped evaluation was the independent variable, and the “likeability of the target” (male evaluator) was the dependent variable. If women in either of the positive stereotyped evaluation conditions liked the male target less than the women in the control condition did, it indicated that the participants disliked being evaluated based on the positive stereotype that women are virtuous. The three mediators were perception of being depersonalized, perception of the competence evaluation of the male class leader toward the participant, perception of family role engagement evaluation that male class leader toward the participant. The effects of these mediators were tested simultaneously.

First, model 1 tested the effect of the independent variable (positive stereotype evaluation) on the dependent variable (likeability of the male class leader); the equation was significant, *R*^2^ = 0.05, *p* = 0.07. Being in the individual positive stereotypical evaluation condition significantly negatively predicted the liking of the male evaluator, *B* = −1.16, *t* = −2.02, *p* = 0.04, and being in the group positive stereotypical evaluation condition did not, *B* = −0.05, *t* = −0.10, *p* = 0.91. The participants in the positive stereotype conditions liked the male class leader less than the participants in the control condition did, indicating that the work-oriented women did not like the person who evaluated them based on the stereotype that women are virtuous.

Then, in the model in which the positive stereotype evaluation affected Mediator 1 (perception of depersonalization), the equation was not significant, *R*^2^ = 0.01, *p* = 0.75. Being in the individual and group positive stereotypical evaluation condition did not significantly predict the perception of being depersonalized, *B* = 0.76, *t* = 0.68, *p* = 0.50; *B* = 0.64, *t* = 0.63, *p* = 0.53. The equation in which the independent variable (positive stereotype evaluation) affected Mediator 2 (competence perception) was not significant, *R*^2^ = 0.03, *p* = 0.26. Being in the individual and group positive stereotypical evaluation condition did not significantly predict the competence perception, *B* = −1.44, *t* = −1.63, *p* = 0.11; *B* = −0.84, *t* = −1.06, *p* = 0.29. The equation in which the independent variable (positive stereotype evaluation) affected Mediator 3 (family-role perception) was significant, *R*^2^ = 0.10, *p* < 0.01. Being in the individual and group positive stereotypical evaluation condition both significantly predicted the family-role perception of the participants, *B* = 4.12, *t* = 2.60, *p* = 0.01; *B* = 4.27, *t* = 3.00, *p* = 0.003. Compared with participants in the control condition, participants in the positive stereotyping condition and the group positive stereotyping condition were more likely to believe that they were perceived as family-oriented in the mind of this male class leader.

In the model of positive stereotype evaluation, the three mediators (feeling of depersonalization, perception of being evaluated as competent, and family-role perception) each predicted the dependent variable (likeability of the male class leader). The equation was significant, *R*^2^ = 0.38, *p* < 0.001. Being in the individual and group positive stereotypical evaluation condition did not significantly predict the likeability of the male class leader, *B* = −0.48, *t* = −0.97, *p* = 0.33; *B* = 0.53, *t* = 1.19, *p* = 0.24. The feeling of depersonalization, perception of being evaluated as competent, and family-role perception each predicted the likeability of the male class leader, *B* = −0.17, *t* = −3.50, *p* < 0.001; *B* = 0.15, *t* = 2.71, *p* = 0.008; *B* = −0.08, *t* = −2.38, *p* = 0.02.

The mediating effect analysis showed that the mediating role of feeling depersonalized in the relationship between the positive stereotype evaluation (dummy variable 1 and dummy variable 2) and the likeability of the male class leader was not significant, *B* = −0.13, *LLCI* = −0.58, *ULCL* = 0.22; *B* = −0.11, *LLCI* = −0.53, *ULCL* = 0.22. The mediation role of the perception of being evaluated as competent was also not significant, *B* = −0.22, *LLCI* = −0.57, *ULCL* = 0.05; *B* = −0.13, *LLCI* = −0.41, *ULCL* = 0.11. Only the mediating role of family-role perception was significant, *B* = −0.33, *LLCI* = −0.78, *ULCL* = −0.02; *B* = −0.34, *LLCI* = −0.77, *ULCL* = −0.03. The evaluation of the male class leader based on the positive stereotype of women being virtuous appeared to make the participants think that they were perceived as family-oriented in the mind of male class leader, leading to the low likeability of male class leader.

## Discussion

The purpose of the current study was to analyze the attitude of women toward the evaluations made by a male based on the positive stereotype that women are virtuous. Our study effectively extends previous studies. First, we found that work-oriented women liked both group-based and individual-based positive stereotype evaluations less than family-oriented women did. Second, we explored the attitude of women toward positive stereotype evaluation from the actor and observer perspectives. Experiment 1 answered the question of whether women like the female target who has been evaluated as virtuous by their male peers. Experiment 2 answered the question of whether women liked the male target who evaluated them (the participant) as virtuous. Third, our study identified family/work centrality as a boundary condition of the link between positive stereotype evaluations and attitude toward those evaluations, partially confirming the assumption that work-oriented women would react negatively to evaluation based on the positive stereotype of women being virtuous. Fourth, we analyzed the internal mechanisms of rejection of positive stereotype evaluation. The positive stereotype evaluation of being virtuous appeared to highlight the implicit requirements of the family role, thus leading to the work-oriented women rejecting evaluation based on this stereotype.

Our study can help people understand attitude of women toward positive stereotyping and thereby enrich models of social cognition and gender role theory. The research results also have certain social practical significance, in that they suggest that making targeted social evaluations according to individual characteristics is helpful for improving interpersonal attraction and reasonable impression management.

### The Boundary of Rejection of Women of the Positive Stereotype Evaluation

We expanded previous research by identifying a boundary of rejection of women of evaluation based on the stereotype that women are virtuous. The study found that family-oriented tendency of the participants was a key moderator of the effect of this type of evaluation on attitudes of women: work-oriented women much more disliked this type of evaluation than family-oriented women did. We suspect this result might be due to the value that the work-oriented female participants place on personal career development and achievement rather than family. The “virtuous” evaluation implies family role demand for women by the society (women should put family first), and this is in conflict with the self-worth of the work-oriented women. Moreover, the virtuous evaluation indicated women would be judged by the traditional gender stereotype, the work-oriented women would be unacceptable and negatively evaluated, and mothers tend to be punished more harshly for tragic instances involving parental neglect. For these reasons, work-oriented women may reject evaluation based on the positive stereotype that women are virtuous.

### Mechanism of Rejection of Evaluations Based on the Stereotype That Women Are Virtuous

We also expanded previous research by identifying a mechanism to explain why work-oriented women reject evaluation based on being virtuous. The results of Experiment 1 showed that work-oriented participants perceived the target of group positive stereotype evaluation and individual positive stereotype evaluation as more family-oriented compared with participants in the control group, which led to work-oriented women reacting negatively to the female target. The results of Experiment 2 indicated that the participants thought the evaluator who gave them a group positive stereotype evaluation or individual positive stereotype evaluation would perceive them to be more engaged in family roles, and they thus disliked the evaluator. In conclusion, the two experiments confirmed that family role perceptions are a key reason for rejection of evaluations made based on the stereotype that women are virtuous for the work-oriented women.

The word “virtuous”(贤惠) is associated with the traditional social demands of family roles and responsibilities. The implicit expectation is that women should not be work-oriented, and should not earn a higher income or social status than men. These expectations constitute a kind of gender discrimination and prejudice. Work-oriented women would hence be likely to reject this form of evaluation.

The mediating role of depersonalization was not confirmed in this study. Experiment 1 assessed depersonalization of the female target, and Experiment 2 tested the feeling of being depersonalized. Neither of these mediated the association between positive stereotype evaluation and the attitude of women toward the evaluation. This may be due to cultural factors, as the study was conducted in China, a collectivist culture. Previous research has indicated that positive stereotype evaluations may cause members of individualistic cultures to feel depersonalized (Siy and Cheryan, 2013). Depersonalization might be experienced as “microaggressions” for those who value personal distinction, and thus women in individualistic cultures may be more likely than those in collectivist cultures to reject such positive stereotype evaluations (Siy and Cheryan, 2013). By contrast, members of collectivist cultures may be more accepting of positive stereotype evaluations (Siy and Cheryan, 2013) due to the fact that people in collectivist cultures have a higher willingness to find a sense of security and group belonging, and are thus less likely to experience depersonalization.

### Limitations and Future Directions

There are several limitations of this study that should be noted. First, a self-administered family orientation questionnaire was used to measure family/work centrality of participants; the continuous variable was then transformed into a categorical variable (family-oriented and work-oriented) with the median as criterion, and family orientation and work orientation were treated as two ends of a continuous variable. However, the constructs represented by these variables are complex (Chang et al., 2010; Beigi et al., 2017). Further research could measure both the work-orientation and family-orientation, with a high family-oriented score and low work-oriented score indicating a family-oriented tendency, and a high work-oriented score and low family-oriented score indicating a work-oriented tendency.

In addition, one-item survey was used to measure the willingness to be the female target. Even though one-item surveys are widely used in social psychology research (Wei and Zhang, 2019), interpretation should be approached with caution. A single-item measure might be adequate when the underlying construct is homogeneous, but multiple-item measures are needed to measure complex constructs reliably. Moreover, we were interested in the attitude of women toward the positive stereotype that women are virtuous, but we measured this indirectly by asking whether they wanted to be like a person who was virtuous, and how much they liked an evaluator who viewed them as virtuous. In future research, the attitude of women toward the virtuous stereotype could be measured more directly.

We used the depersonalized perception scale, which has been used in previous research, to analyze the mediation role of depersonalization in the relation between the positive stereotype evaluation and the attitude of women toward the evaluation. However, the internal consistency of this scale was low both in the previous study and our study. We suspected that the complicated wording of the items may make it difficult for some participants to fully understand their meaning. This may have contributed to the non-significant mediation effect of this variable. Future research should test other methods of measuring the perception of depersonalization.

The mediator effect of the competence perception was also non-significant. We believe this non-significant result might have been obtained because the context in our study did not involve a directly competence evaluation, only the guess about what the evaluation would be. The parallel constraint satisfaction model suggests that different context information activates different subsets of a network of connections, and motives and objectives change across contexts (Crisp and Hewstone, 2006). It may be that in a competence-related situation, women are more resistant to the virtuous evaluation, whereas in a family-related situation, people are more inclined to the virtuous evaluation. Therefore, future research needs to explore the effect of context on attitudes toward positive stereotype evaluations.

In our study, women were described as being evaluated as virtuous by a male peer. In this respect, the woman could be considered part of the in-group and the male evaluator could be considered part of the out-group. Social identity theory holds that people identify with their own group, and individuals could improve their self-esteem by achieving or maintaining a positive social identity (Scheifele et al., 2020). We tend to view in-group members as diverse and non-stereotyped, and evaluations by in-group members are less likely to be perceived as bias or discrimination. However, evaluations by out-group members are more biased, and a positive stereotype evaluation from someone in the out-group (e.g., Czopp, 2008; Siy and Cheryan, 2013) is more easily identified as prejudiced, hostile, and problematic (Kilianski and Rudman, 1998). Thus, a positive stereotype evaluation by in-group and out-group members may have different results, and future research could focus on this issue.

## Data Availability Statement

The raw data supporting the conclusions of this article will be made available by the authors, without undue reservation.

## Ethics Statement

The studies involving human participants were reviewed and approved by the Institution of Psychology in China University of Geosciences. The participants provided their written informed consent to participate in this study.

## Author Contributions

JS and YR designed the experiment and wrote the manuscript. YL and JL collected the data and analyzed the data. All authors contributed to the article and approved the submitted version.

## Conflict of Interest

The authors declare that the research was conducted in the absence of any commercial or financial relationships that could be construed as a potential conflict of interest.

## Publisher’s Note

All claims expressed in this article are solely those of the authors and do not necessarily represent those of their affiliated organizations, or those of the publisher, the editors and the reviewers. Any product that may be evaluated in this article, or claim that may be made by its manufacturer, is not guaranteed or endorsed by the publisher.
